# Inhibition of insulin-like growth factor receptor/AKT/mammalian target of rapamycin axis targets colorectal cancer stem cells by attenuating mevalonate-isoprenoid pathway *in vitro* and *in vivo*

**DOI:** 10.18632/oncotarget.3684

**Published:** 2015-03-29

**Authors:** Chetna Sharon, Somesh Baranwal, Nirmita J. Patel, Daniel Rodriguez-Agudo, William M. Pandak, Adhip PN Majumdar, Geoffrey Krystal, Bhaumik B. Patel

**Affiliations:** ^1^ Hunter Holmes McGuire VA Medical Center, Richmond, VA, USA; ^2^ Massey Cancer Center, Virginia Commonwealth University, Richmond, VA, USA; ^3^ Division of Hematology, Oncology and Palliative Care, Virginia Commonwealth University, Richmond, VA, USA; ^4^ Department of Medicine, Division of Gastroenterology, Virginia Commonwealth University, Richmond, VA, USA; ^5^ John D. Dingell VAMC, Detroit, MI, USA

**Keywords:** cancer stem cells, insulin-like growth factor-1 receptor, mTOR, mevalonate pathway, isoprenoids

## Abstract

We observed a co-upregulation of the insulin-like growth factor receptor (IGF-1R)/AKT/mammalian target of rapamycin (mTOR) [InAT] axis and the mevalonate-isoprenoid biosynthesis (MIB) pathways in colorectal cancer stem cells (CSCs) in an unbiased approach. Hence, we hypothesized that the InAT axis might regulate the MIB pathway to govern colorectal CSCs growth. Stimulation (IGF-1) or inhibition (IGF-1R depletion and pharmacological inhibition of IGF-1R/mTOR) of the InAT axis produced induction or attenuation of CSC growth as well as expression of CSC markers and self-renewal factors respectively. Intriguingly, activation of the InAT axis (IGF-1) caused significant upregulation of the MIB pathway genes (both mRNA and protein); while its inhibition produced the opposite effects in colonospheres. More importantly, supplementation with dimethylallyl- and farnesyl-PP, MIB metabolites downstream of isopentenyl-diphosphate delta isomerase (IDI), but not mevalonate and isopentenyl-pp that are upstream of IDI, resulted in a near-complete reversal of the suppressive effect of the InAT axis inhibitors on CSCs growth. The latter findings suggest a specific regulation of the MIB pathway by the InAT axis distal to the target of statins that inhibit 3-hydroxy-3-methyl-glutaryl-CoA reductase (HMGCR). Effects of IGF-1R inhibition on colonic CSCs proliferation and the MIB pathway were confirmed in an ‘*in vivo*’ HCT-116 xenograft model. These observations establish a novel mechanistic link between the InAT axis that is commonly deregulated in colorectal cancer and the MIB pathway in regulation of colonic CSCs growth. Hence, the InAT-MIB corridor is a novel target for developing paradigm shifting optimum anti-CSCs therapies for colorectal cancer.

## INTRODUCTION

Despite a significant improvement in the cancer therapeutic armamentarium in the past decade, the outcomes for colorectal cancer remain poor as disease relapse following standard treatment is a common phenomenon [1]. Increasing literature suggests that most epithelial tumors are composed of heterogeneous population of cells with differing tumorigenic potential organized in a hierarchical fashion [2-4]. In colorectal cancers, a relatively rare population of cells that expresses distinct set of cell surface markers, such as CD133, CD44, C-X-C chemokine receptor type 4 (CXCR4) and Leucine-rich repeat-containing G-protein coupled receptor 5 (LGR5), have been identified as highly tumorigenic and are referred to as cancer stem cells (CSCs) [5, 6]. An emerging therapeutic paradigm suggests that targeting CSCs, that survive initial chemotherapy or radiation insult resulting in disease relapse, is likely to overcome shortcomings of current anti-cancer therapies [2, 7].

Emerging literature highlights the role of Insulin-like growth factor (IGF)-1 receptor (IGF-1R) pathway in regulating ‘CSC properties’ in several epithelial cancers, including colorectal cancer [8, 9]. In fact, anti-IGF-1R therapies have been shown to inhibit CSCs growth and overcome resistance to chemotherapy and/or radiation [10, 11]. The binding of IGFs to IGF-1R results in receptor autophosphorylation and activation of critical downstream targets, including the PI3K/AKT/mTOR signaling (InAT axis) as well as RAS/RAF/MAPK pathways [12]. Of these, the InAT axis is particularly linked with regulating stemness in a variety of cancer models [13, 14]. In addition to IGF-1R, AKT and mTOR have also been shown to play a critical role in breast CSCs [13, 14]. However their role in colon CSCs is not well studied. Sadly, the hype related to anti-IGF-1R therapies have not translated into clinical benefit due to a variety of reasons, including a poor understanding of the detailed mechanism of action of anti-IGF-1R therapy on CSC growth [15].

The mevalonate-isoprenoid biosynthesis (MIB) pathway (refer to Fig. 5) is involved in production of farnesyl pyrophosphate (FPP), which regulates critical biological processes such as hormone synthesis, N-glycosylation and protein prenylation amongst others, from acetyl-CoA, an intermediary metabolite [16]. Mevalonic acid (MVA), the synthesis of which is a rate limiting step in the pathway, and isoprenes such as isopentenyl pyrophosphate (IPP) and dimethylallyl pyrophosphate (DMAPP) are important intermediates in this pathway. Therefore, this pathway will be referred to as the MIB pathway [17, 18] henceforth. Hydroxymethylglutaryl coenzyme A reductase (HMGCR), the rate limiting enzyme of the MIB pathway, induced neoplastic transformation of MCF 10A mammary epithelial as well as promoted the growth of transformed MCF7 breast cancer cells [19], highlighting the importance of the MIB pathway in carcinogenesis. This pathway is particularly activated by gain-of-function mutations in p53 gene and also plays a very important role in the breast CSCs growth [20, 21]. Recently, the MIB pathway has generated a remarkable interest as a therapeutic target, partly due to the fact that it is readily inhibited by several FDA approved drugs or combinations thereof [16, 22].

In the current manuscript, we demonstrate a novel role of IGF-1R in regulating CSCs growth through modulation of isoprenoid biosynthetic pathway *in vitro* and *in vivo*. Specifically, we have uncovered isopentenyl diphosphate delta isomerase (IDI) as a target of IGF-1R regulation. These results suggest that inhibition of the MIB pathway by IGF-1R inhibitor is at a distinct level than that targeted by statins or nitrosylated bisphosphonates, which inhibit HMGCR and farnesyl diphosphate synthase (FDPS) respectively. These findings have essential therapeutic implications for colorectal cancer in particular and IGF-1R responsive cancer in general.

## RESULTS

### Activation of InAT axis is observed in colorectal CSCs

**Figure 1:**
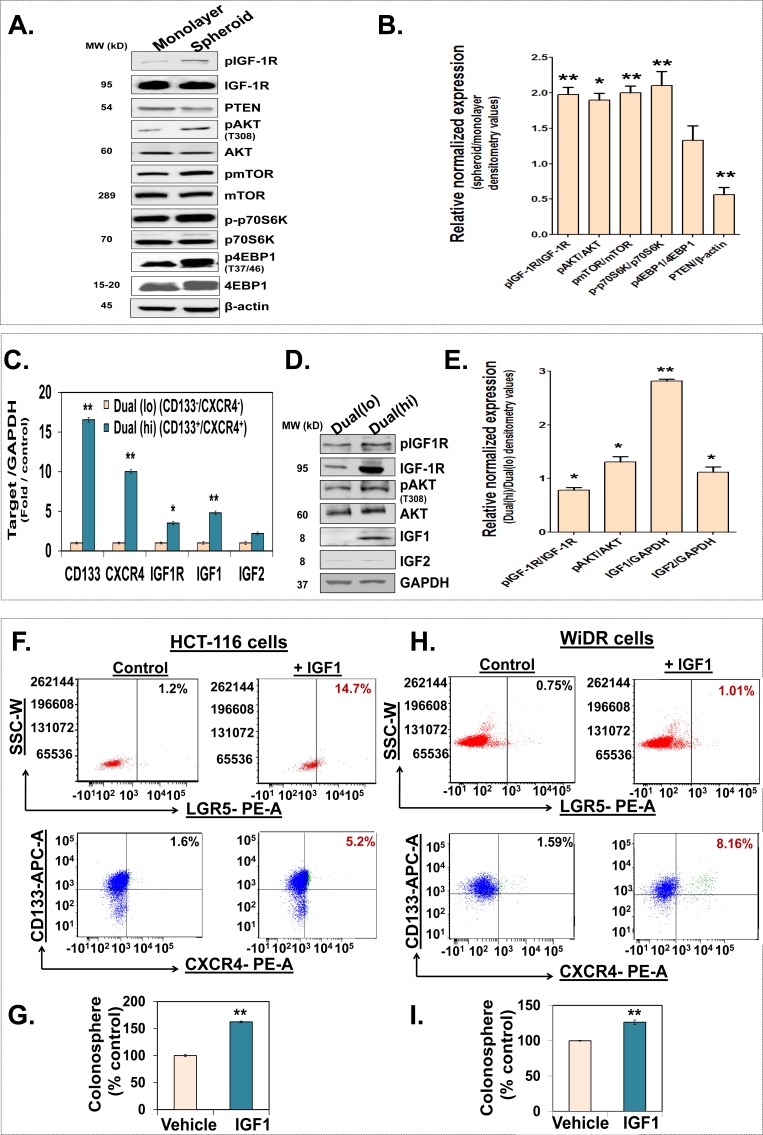
Activation of the InAT axis is observed in colorectal CSCs (**A** and **B**) Representative immunoblots and the corresponding bar graph of the gel densitometry analyses of relative normalized expression of the InAT axis proteins including activated (phosphorylated) levels of IGF-1R, AKT, mTOR, p70S6K and 4EBP1 (normalized to total protein expression) as well as PTEN, an inhibitor of InAT axis, in HCT-116 spheroids compared to respective monolayer controls. β-actin is used as a loading control. (**C**) Q-PCR analyses demonstrating differential expression of IGF-1R pathway genes and CSC markers in Dual(hi) [CD133(hi)/CXCR4(hi), CSCs] compared to Dual(lo) [CD133(low)/CXCR4 (low), non-CSCs] cells. Data was normalized to GAPDH. (**D** and **E**) Representative immunoblots and the corresponding bar graph of the gel densitometry analyses show higher expression of phosphorylated IGF-1R and AKT (normalized to total protein expression) as well as substantial increase in IGF1 level in Dual(hi) HT-29 cells compared to Dual(lo) controls. GAPDH is used as a loading control. (**F** and **H**) HCT-116 and WiDR cells grown in monolayer condition were serum starved for 24 h followed by stimulation with IGF1 (100 ng/ml) for 48 hours. Representative scattered plots of flow cytometry analyses for LGR5 positive and Dual(hi) cells (CD133 positive cells depicted in blue and CXCR4 positive cells depicted in green) show increase in proportion of CSCs in IGF1 stimulated cells compared to unstimulated controls. (G and I) Colonosphere formation in HCT-116 and WiDR monolayer cells stimulated with IGF1 (100 ng/ml) compared to respective controls. Data are presented as mean ± SD (*n* = 3). *p < 0.05; **p < 0.005.

We and others have shown that IGF-1R is activated in chemoresistant/chemosurviving cells as well as side population (SP) fraction of colorectal cells that are enriched in CSCs [8, 10, 23, 24] To further elucidate the role of IGF-1R in CSCs, we examined activation of IGF-1R pathway in CSCs enriched spheroids [25, 26]. We observed a significant increase in not only the total IGF-1R but also its activated form pIGF-1R as well as downstream mediators, including activated forms (phosphorylated) of AKT, mTOR, p70S6K and 4EBP1 in HCT-116 spheroids compared to their monolayer counterparts (Fig. 1A & 1B). Moreover, levels of PTEN, an inhibitor of this pathway, were significantly downregulated in the spheroids (Fig 1A & 1B). Although, the spheroid cultures are enriched in CSCs, they represent a mixture of stem and progenitor cells [27]. We pursued further enrichment of CSCs through fluorescent assisted cell sorting (FACS) with two colonic CSCs markers CD133 and CXCR4 in HT-29 spheroid cells. Indeed, CD133(hi)/CXCR(hi) cells, also Dual (hi), showed approximately 3-fold increase in spheroid formation compared to CD133(low)/CXCR4(low), also Dual (lo), controls; suggesting that Dual (hi) cells represent CSCs (Supplementary Fig. 1). Upon further analyses, we observed approximately 3- to 4-fold increase in IGF-1R mRNA and protein levels in Dual (hi) (CSCs) compared to Dual (lo) (non-CSCs) (Fig. 1C, 1D & 1E). Additionally, we observed a significant increase in IGF-1 but not IGF-2 mRNA (5-fold) and protein (~ 2.8-fold) levels in Dual (hi) cells compared to the controls (Fig. 1C & 1D). Importantly, we observed a concomitant increase in pIGF-1R (1.6-fold) as well as its downstream mediator pAKT (2-fold) in Dual (hi) cells compared to Dual (lo) controls (Fig. 1D). The latter findings are consistent with the increased levels of IGF-1 (ligand) and IGF-1R (receptor) in Dual (hi) cells. As these changes were observed in non-stimulated condition, we sought to determine the effect of IGF-1 stimulation on the proportion of CSCs and their growth. HCT-116 and WiDR monolayer cells that were treated with IGF-1 (100 ng/ml) for 48 h following overnight serum starvation, showed a significant increase in proportion of LGR5 positive, an established colon CSC marker [5], as well as Dual (hi) cells (Fig. 1F & 1H). Moreover, there was a significant increase in spheroid formation in IGF-1 stimulated cells compared to vehicle treated controls (Fig. 1G & 1I); suggesting that colon cancer cells respond to IGF-1 stimulation by increasing proportion of CSCs. Overall, we observed a significant activation of InAT axis in colorectal CSCs.

### Inhibition of IGR-1R, an upstream regulator of InAT axis, suppresses colon CSCs growth and self-renewal

**Figure 2:**
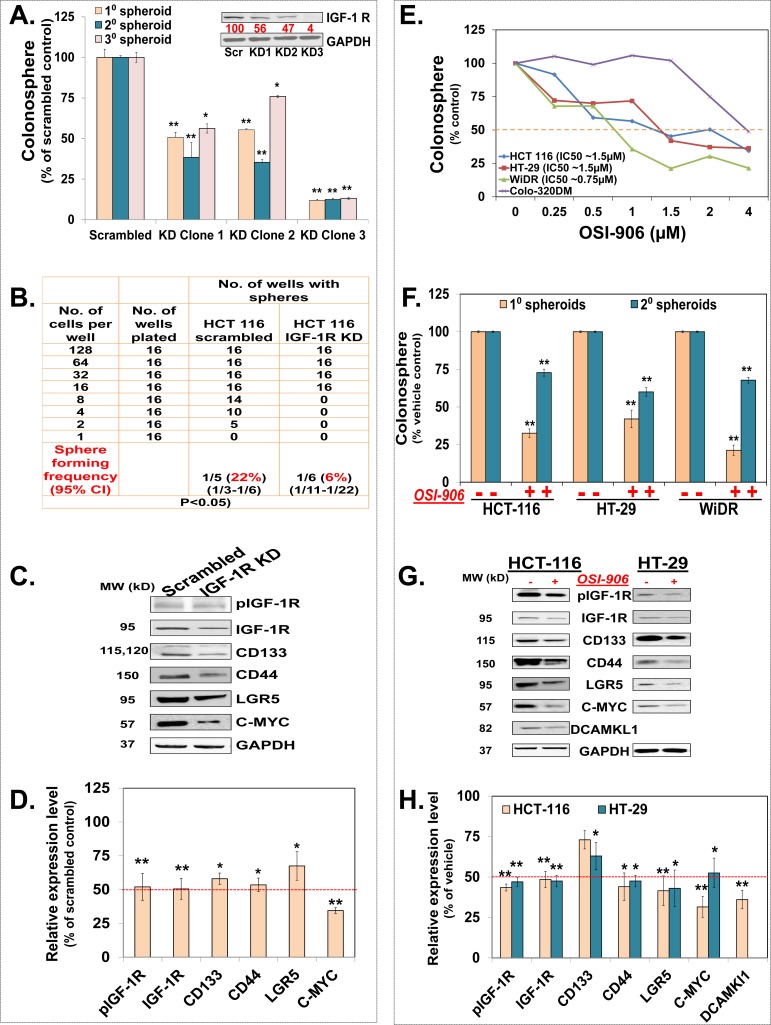
Inhibition of IGR-1R, an upstream regulator of the InAT axis, suppresses colon CSCs growth and self-renewal (**A**) Immunoblot analyses show reduced IGF-1R expression and corresponding inhibition of 1^o^/2^o^/3^o^ spheroid formation in different subclones of HCT-116 cells stably transfected with IGF-1R shRNA compared to scrambled controls; suggesting attenuation of CSC growth and self-renewal by IGF-1R gene knockdown. (**B**) Extreme limiting dilution assay shows reduced spheroid forming frequency of HCT-116 cells transfected with IGF-1R shRNA (Clone 2, IGF-1R KD) compared to scramble transfected control. (**C & D**) Representative immunoblots and the corresponding bar graph of the gel densitometry analyses show decreased expression of activated and total IGF-1R as well as CSC markers (CD133, CD44, and LGR5) and self-renewal factor (C-MYC) in IGF-1R KD spheroids compared to scrambled controls. (**E**) Dose response curve shows dose dependent inhibition of primary colonosphere formation in three out of four colon cancer cell lines treated with OSI-906, a tyrosine kinase inhibitor of IGF-1R, at an IC_50_ ranging from 0.75 μM −1.5 μM compared to vehicle treated control. (**F**) OSI-906 (1.5 μΜ) inhibits 1^o^ as well as 2^o^ spheroid formation in HCT-116, HT-29 and WiDR colon cancer cells suggesting attenuation of CSC growth and self-renewal. (**G & H**) Representative immunoblots and the corresponding bar graph of the gel densitometry analyses show decreased expression of activated IGF-1R (at 15 min) as well as CSC markers (CD133, CD44, LGR5, and DCAMKL1) and self-renewal marker (C-MYC) (24 hours) in HCT-116 and HT-29 spheroids treated with OSI-906 (1.5 μΜ) compared to vehicle treated controls. GAPDH is used as loading control for all immunoblot analyses. Data are presented as mean ± SD (*n* = 3). Numbers under the blot represent relative densitometry values. *p < 0.05; **p < 0.005.

In order to determine the functional significance of activation of IGF-1R pathway in colon CSCs, we examined the effects of depletion (shRNA) of or pharmacological inhibition of IGF-1R (tyrosine kinase inhibition; OSI-906) on CSCs growth. We generated cells stably expressing reduced levels of IGF-1R by shRNA transfection and subsequent selection with puromycin. Clones showing modest depletion of IGF-1R levels (47-56%) caused a similar 40-50% inhibition in the colonosphere formation (Fig. 2A) compared to scrambled shRNA transfected controls. Likewise, the clones exhibiting a robust (>90%) inhibition of IGF-1R caused an equally strong (>90%) inhibition in the colonosphere formation compared to scrambled controls (Fig. 2A); suggesting a critical role for IGF-1R in regulating CSCs growth. We used KD clone 2 that shows ~50% reduction (pharmacologically relevant inhibition) in IGF-1R levels for all the future experiments. Limiting dilution assay using this clone shows an approximately 3-fold reduction in spheroid forming capacity compared to controls (Fig. 2B). Moreover, IGF-1R KD cells show continued inhibition of CSCs growth during subsequent propagation in SCM (2° & 3° spheroids), suggesting its role in regulating CSCs self-renewal (Fig. 2A). More importantly, IGF-1R KD spheroids show significant reduction in levels of several CSC markers such as CD133, CD44 and LGR5 (25-43%) as well as self-renewal factor C-MYC (74%) compared to scrambled transfected controls (Fig. 2C & 2D). Encouraged by the results with specific knockdown of IGF-1R, we examined the effect of OSI-906, a clinically relevant tyrosine kinase inhibitor (TKI) of IGF-1R, on CSCs growth. We observed a dose dependent inhibition in colonosphere formation in 3 out of 4 colorectal cancer cells with an apparent IC50 (0.75-1.5 μM) that lies in clinically achievable range (Fig. 2E). Moreover, treatment with OSI-906 (1.5 μM) resulted in inhibition of not only 1° spheroids but also 2° spheroids, derived from the single cell suspension of the 1° spheroids and propagated in SCM media without any further treatment, in all three colorectal cancer cells (Fig. 2F), suggesting inhibition of CSC self-renewal. Finally, in support of the phenotypic effects on CSCs growth by OSI-906, we observed a significant reduction in the expression of several CSCs markers including CD133 (23-43%), CD44 (55-62%), LGR5 (49-52%), and doublecortin and CaM kinase-like 1 (DCAMKL1) (60%), as well as self-renewal factor C-MYC (41-73%) (25) in both HCT-116 and HT-29 colon cells (Fig. 2G & 2H). Overall, the above findings unequivocally suggest that inhibition of IGF-1R attenuates CSC properties and that these effects can be achieved with clinically relevant concentrations of IGF-1R TKI.

### Mevalonate-Isoprenoid biosynthesis (MIB) pathway is upregulated in colon CSCs

**Figure 3:**
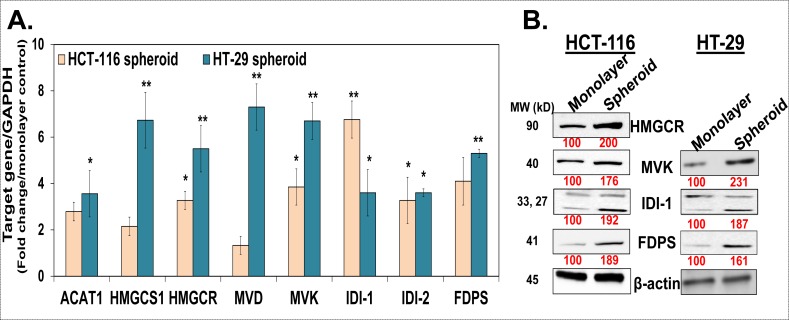
The mevalonate-isoprenoid biosynthesis (MIB) pathway is upregulated in colon CSCs (**A**). Q-PCR analyses show higher expression of several MIB pathway genes in HCT-116 and HT-29 spheroids compared to monolayer controls suggesting upregulation of MIB pathway in colorectal CSCs. Data was normalized to GAPDH. (**B**). Immunoblot analyses show increased expression of key MIB pathway proteins in both HCT-116 and HT-29 spheroids compared to monolayer controls. β-actin is used as loading control. Data are presented as mean ± SD (*n* = 3). Numbers under the blot represent relative densitometry values. *p < 0.05; **p < 0.005.

In order to understand the detailed mechanisms of IGF-1R inhibition mediated attenuation of CSCs growth, we focused our attention on identifying a major co-upregulated pathway in colon CSCs. To this end, we performed a gene microarray analysis in HT-29 cells grown as monolayer (non-CSC control) and spheroid (CSCs) using the Affymetrix® platform. Unbiased analyses for the top canonical pathway using the IPA® software revealed that two related pathways, superpathway of the cholesterol biosynthesis and the MIB pathway were amongst the top 7 pathways that were enriched in spheroids compared to monolayer controls (Supplementary Fig. 2). Additionally, IGF-1 signaling pathway was also one of the most enriched pathways in HT-29 spheroids, suggesting co-upregulation of IGF-1 signaling and the MIB pathway. Herein, the MIB pathway is of particular interest given its role in breast CSCs growth [21]. We systemically examined mRNA and/or protein levels of enzymes involved in the MIB pathway in HCT-116 and HT-29 spheroids. We observed a significant increase in the mRNA levels of most of the enzymes in the MIB pathway in spheroids compared to respective monolayer controls (Fig. 3A). However, a few of them such as HMGCR, MVK, IDI (both isoforms) and FDPS showed at least 3-fold increase in mRNA levels in both HCT-116 and HT-29 spheroids (Fig. 3A). We confirmed a similar increase in protein levels (~1.8- to 2.3-fold) of these enzymes in HCT-116 and HT-29 spheroids compared to controls (Fig. 3B). Hence, the MIB pathway is co-upregulated with IGF-1R pathway/InAT axis in colorectal CSCs.

### The InAT axis regulates the MIB pathway in CSCs ‘*in vitro*’

**Figure 4:**
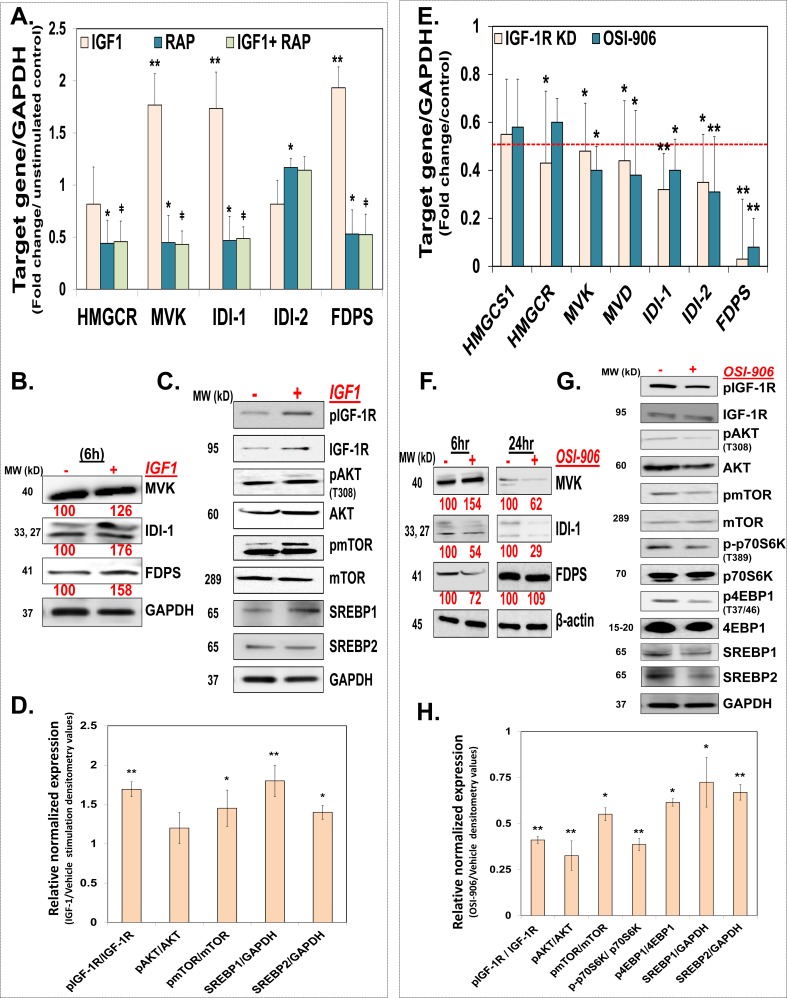
The InAT axis regulates the MIB pathway in CSCs ‘*in vitro*’ (**A**) Q-PCR analyses show increased expression of MIB pathway genes in HCT-116 spheroids stimulated with IGF1 (10 ng/ml) for 6 h compared to unstimulated controls. Moreover, pretreatment with Rapamycin (3 nM) abrogates increase in MIB pathway gene expression seen with IGF-1 stimulation. Data was normalized to GAPDH. **p < 0.005 compared to vehicle treated control; ǂ p < 0.05 compared to IGF1 treated spheroid. (**B**) Immunoblots showing increased expression of select MIB pathway proteins in HCT-116 spheroids stimulated with IGF1 (10 ng/ml) for 6 hours compared to controls. GAPDH is used as loading control. (**C** and **D**) Representative immunoblots and the corresponding bar graph of the gel densitometry analyses in HCT-116 spheroids stimulated with IGF1 (10 ng/ml) show increased expression of pIGF-1R and downstream mediator pAKT and pmTOR (at 15 min) (normalized to total protein expression) as well as increased expression of cleaved form of SREBPs (transcriptionally active (at 6 hours)), compared to unstimulated controls. GAPDH is used as loading control. (**E**) Q-PCR analyses showing decreased expression of MIB pathway genes in IGF-1R KD, as well as HCT-116 spheroids treated with OSI-906 (1.5 μΜ) for 24 h compared to scrambled or vehicle treated controls respectively. Data was normalized to GAPDH. (**F**) Immunoblot analyses show varying degree of inhibition of select MIB pathway proteins at early (6 h) and late (24 h) time points following OSI-906 (1.5 μΜ) treatment in HCT-116 spheroids. β-actin is used as loading control. (G and H) Representative immunoblots and the corresponding bar graph of the gel densitometry analyses show decreased levels of activated form of IGF-1R as well as downstream components of InAT axis (at 15 min) (normalized to total protein expression), as well as cleaved form of SREBP2 (transcriptionally active (1 h treatment)) in OSI-906 (1.5 μM) treated HCT-116 spheroids compared to vehicle treated controls. GAPDH is used as loading control. Numbers under the blot represent relative densitometry values. Data are presented as mean ± SD (*n* = 3). *p < 0.05; **p < 0.005.

To determine whether activation of InAT axis regulates the MIB pathway in CSCs, we treated HCT-116 spheroids with IGF-1 (10 ng/ml) or vehicle and examined the expression of the MIB pathway enzymes at mRNA and protein levels. We observed a significant increase in the mRNA (~2- to 3.5-fold) and protein (~1.3- to 2.8-fold) levels of MVK, IDI (both isoforms) and FDPS compared to vehicle treated controls (Fig. 4A & 4B). On the other hand, levels of HMGCR, a target of statins, were not increased further by IGF-1 stimulation, suggesting that major regulation of the MIB pathway by IGF1/IGF-1R might be distal to MVA production (Fig. 4B). Consistent with the earlier observation that mTOR singling is activated downstream of IGF-1R activation in CSCs (Fig. 1A & 1B), IGF-1 treatment in HCT-116 spheroids caused increased levels of the activated form of not only IGF-1R (~2.2-fold) but also AKT (~1.8-fold) and mTOR (1.35-fold) compared to vehicle treated controls (Fig. 4C & 4D). In order to understand the functional significance of mTOR activation on regulation of the MIB pathway, we examined the effects of rapamycin, a known inhibitor of mTORC1 complex, on abrogating IGF-1 mediated induction of the MIB pathway genes. Indeed, rapamycin treatment alone was effective in inhibiting expression of several of the MIB pathway genes (Fig. 4A). More importantly, rapamycin pretreatment reversed the effect of IGF-1 on induction of MVK, IDI-1 and FDPS mRNA levels (Fig. 4A). Additionally, rapamycin treatment also inhibited spheroid growth in a dose dependent fashion (Supplementary Fig. 3). To further determine the regulatory role of IGF-1R activation on the MIB pathway, we examined the effect of IGF-1R inhibition (both genetic and pharmacological with OSI-906) on expression of the MIB pathway enzymes at mRNA and protein levels in HCT-116 spheroids. Firstly, as expected, OSI-906 treatment resulted in inhibition of not only pIGF-1R but also downstream mediators, including pAKT, pmTOR, p70S6K and p4EBP1 in HCT-116 spheroids (Fig 4G & 4H). Moreover, we observed a significant (>50%) reduction in the mRNA levels of HMGCS1, HMGCR, MVK, IDI (both isoforms) and FDPS by IGF-1R depletion and OSI-906 treatment (Fig. 4E). However, the effect on IDI- (1&2) and FDPS was more pronounced in response to IGF-1R inhibition (Fig. 4E), supporting the notion that major regulation of the MIB pathway by IGF-1R might be distal to MVA production. Interestingly, at the protein level while OSI-906 treatment caused early (6 hours) and sustained (24 hours) reduction in IDI-1 levels, it only caused brief (FDPS) or late (MVK) inhibition of other MIB pathway genes (Fig. 4F).

Several reports suggest that IGF1/IGF-1R might regulate steroid biosynthesis by promoting cleavage of sterol regulatory element binding proteins (SREBP) and its subsequent translocation to the nucleus [28]. Of the two major isoforms of SRBEBPs, SREBP2 is especially critical in regulation of the MIB pathway gene expression(29). Importantly, activation of AKT and/or mTOR plays an important role in mediating SREBP cleavage by IGF-1 [28, 30]. We observed increased levels of cleaved (also mature) form of SREBPs (1&2) in response to IGF1 stimulation (Fig. 4C). On the other hand, OSI-906 treatment caused decreased expression of mature forms of SREBPs (1 & 2) (Fig. 4G). Overall, the above findings suggest that the InAT axis plays an important role in positive regulation of the MIB pathway distal to MVA production, perhaps via down regulation of AKT-mTOR signaling pathway resulting in alteration in SREBP activation in CSCs [30].

### Downregulation of the MIB pathway is required for InAT axis inhibition mediated attenuation of CSCs growth

**Figure 5:**
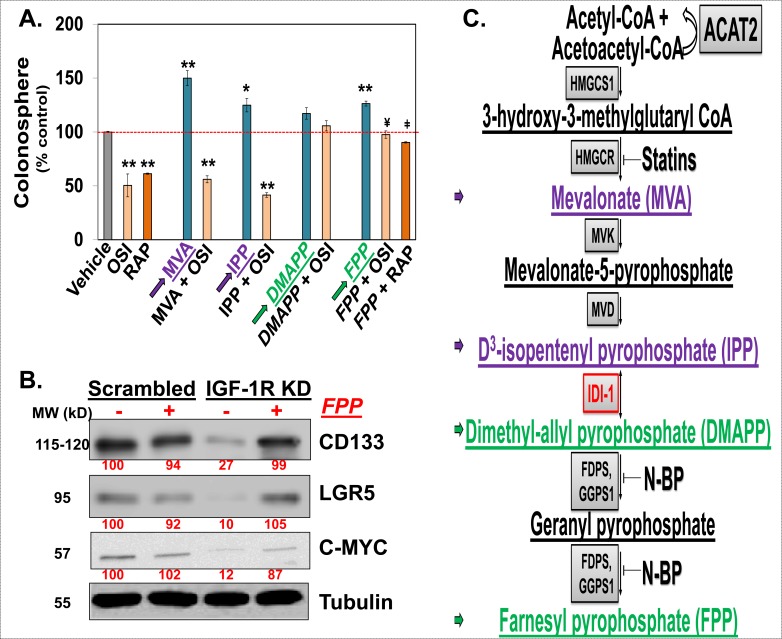
Downregulation of the MIB pathway is required for the InAT axis inhibition mediated attenuation of CSCs growth (**A**): Colonosphere formation assays in HCT-116 cells treated with either OSI-906 (1.5 μM) or Rapamycin (3 nM) alone or along with various metabolites of the MIB pathway (1 μM) show near complete reversal of OSI-906 (and/or Rapamycin) mediated inhibition of spheroid formation by DMAPP and FPP (downstream of IDI-1, highlighted with green arrow) but not by MVA or IPP (upstream of IDI-1, highlighted with purple arrow), compared to vehicle treated control. *p < 0.05, **p < 0.005 compared to vehicle treated control; ¥ *p* < 0.05 compared to OSI-906 treated spheroid; ¥ *p* < 0.005 compared to Rapamycin treated spheroids. (**B**) Immunoblots showing reduced expression of CSC markers (CD133 and LGR5) and a self-renewal factor (C-MYC) in IGF-1R KD cells which is almost completely reversed by supplementation with FPP (10 μM) for 48 hours, strongly suggesting critical role of MIB pathway in mediating IGF-1R's effect on expression of CSC markers. Tubulin was used as loading control. (**C**) Schematic representation of the MIB pathway. Metabolites upstream of IDI-1 are highlighted in purple and downstream of IDI-1 are highlighted in green. Two classes of FDA-approved agents, statins and nitrosylated-bisphophonates (N-BPs), target different enzymes in the MIB pathways than that targeted by InAT axis. Numbers under the blot represent relative densitometry values. Data are presented as mean ± SD (*n* = 3).

To determine if downregulation of the MIB pathway is important for IGF-1R's effect on CSCs growth, we examined the effect of supplementation with several of the MIB pathway metabolites on OSI-906 mediated inhibition of CSCs growth in HCT-116 cells. All the metabolites tested here caused a modest increase in spheroid formation compared to vehicle treated controls. However, mevalonic acid (MVA) and isopentenyl-pyrophosphate (IPP) had no effect, while equimolar concentration of dimethylallyl-pyrophosphate (DMAPP) and farnesyl-pyrophosphate (FPP) completely reversed the OSI-906 mediated inhibition of spheroid formation (Fig. 5A & 5B). Additionally, FPP supplementation also near-completely reversed the effect of rapamycin on spheroid formation, suggesting that IGF-1R signaling through mTOR pathway regulates the MIB pathway and CSCs growth. Moreover, FPP supplementation near-completely reversed IGF-1R KD mediated inhibition in the expression of CSC makers (CD133 and LGR5) and self-renewal factor (C-MYC) (Fig. 5B). Hence, suppression of the MIB pathway downstream of IPP production is critical for the effect of the InAT axis inhibition on attenuation of CSCs growth.

### OSI-906 inhibits the MIB pathway and CSCs ‘*in vivo*’

**Figure 6:**
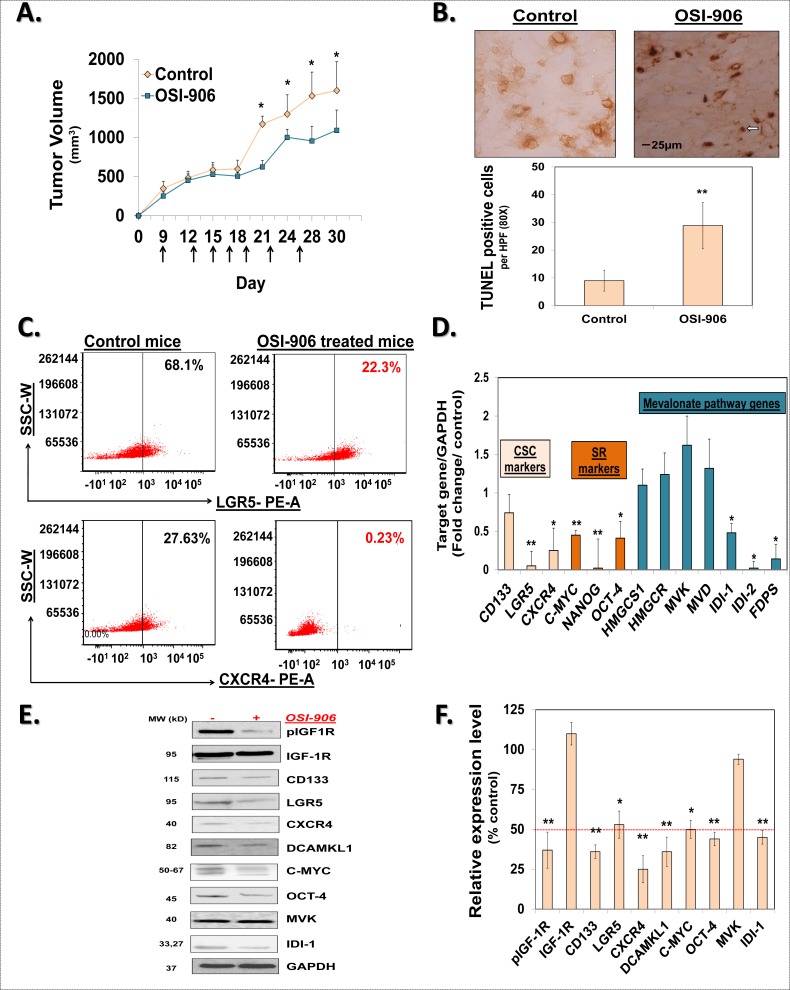
OSI-906 inhibits the MIB pathway and CSCs ‘*in vivo*’ (**A**) Change in mean tumor volume of HCT-116 xenograft in mice treated with oral gavage of OSI-906 or vehicle (control). Arrows indicate the days of OSI-906 / vehicle gavage. (**B**) TUNEL staining for apoptosis in HCT-116 xenografts. Arrow indicates an area of positive staining. Average number of TUNEL-positive cells per HPF (80x) shows significant induction of apoptosis in xenograft derived from mice treated with OSI-906, compared to vehicle treated controls. (**C**) Representative scattered plots of flow cytometry analyses show decrease in expression of CSC markers (LGR5 and CXCR4) in xenograft derived from OSI-906 treated mice, compared to vehicle treated controls. (**D**) Q-PCR analyses showing significant reduction in the mRNA levels of several CSC markers (CD133, LGR5 and CXCR4), self-renewal factors (C-MYC, NANOG, OCT-4) and select MIB pathway genes in OSI-906 treated mouse xenograft tissue compared to controls. Data was normalized to GAPDH. (**E** and **F**) Representative immunoblots and the corresponding bar graph of the gel densitometry analyses show decreased expression of phosphorylated IGF-1R, CSC markers (CD133, LGR5, CXCR4, and DCAMKL1), self-renewal factor (C-MYC and OCT-4) and an MIB pathway protein (IDI-1) in OSI-906 treated xenografts compared to vehicle controls. GAPDH is used as a loading control. Data are presented as mean ± SD (*n* = 3). *p < 0.05; **p < 0.005.

To further validate the role of IGF-1R in regulation of the MIB pathway and CSCs growth *in vivo*, we treated mice bearing HCT-116 xenograft with oral gavages of OSI-906 (25 mg/kg/day) or vehicle, as indicated in Fig. 6A. OSI-906 treatment resulted in a moderate reduction in tumor volume 21-day post treatment initiation (Fig 6A). We performed additional mechanistic studies to. better understand the effect of OSI-906 treatment on CSCs growth. Previous report indicated induction of apoptosis following IGF-1R inhibition [31]. We observed a marked induction of apoptosis as measured by TUNEL-positive cells in OSI-906 treated xenografts consistent with reduction in tumor growth (Fig. 6B). In contrast to moderate tumor volume changes, we observed a robust reduction in proportion of LGR5+ (3-fold) as well as CXCR4+ cells in OSI-906 treated xenografts compared to vehicle treated controls (Fig. 6C). Moreover, we observed a significant reduction in levels of CSCs makers (CD133, LGR5, and CXCR4) as well as self-renewal factors (C-MYC, OCT-4, and NANOG), confirming the OSI-906 mediated inhibition of CSCs growth and self-renewal ‘*in vivo*’(Fig. 6D, 6E & 6F; Supplementary Fig. 4). More importantly, we observed a robust (>50%) inhibition of IDI-1 mRNA and protein levels as well as mRNA expression of IDI-2 and FDPS; while the levels of enzymes upstream of IPP production were largely unaffected (Fig. 6D & 6E). These results strongly suggest that OSI-906 inhibits the MIB pathway downstream of IPP production consequently affecting CSCs growth *in vivo*.

In summary, we demonstrate that InAT axis regulates CSCs growth both *in vitro* and *in vivo*. Moreover, our data for the first time demonstrated that InAT axis's effect on CSCs is dependent on its robust and specific regulation of the MIB pathway genes. These findings have immediate and key therapeutic implications for treatment of colorectal cancer in particular and IGF-1R responsive cancer in general.

## DISCUSSION

Recently the MIB pathway has attracted a lot of attention as a target for development of novel anticancer therapies [16]. Besides its importance in cholesterol biosynthesis, protein prenylation, and N-glycosylation; it plays a critical role in modulating anticancer immune response, highlighting its paramount importance in carcinogenesis [16]. Moreover, the MIB pathway plays a critical role in regulating breast CSCs growth, especially in triple negative breast cancers [21]. Our data clearly support a similar argument for colorectal cancer CSCs. We observed that MIB and super-pathway of cholesterol biosynthesis were amongst the top five canonical pathways that were upregulated in CSCs enriched spheroids compared to monolayer control. Hence, targeting the MIB pathway is expected to selectively target colorectal CSCs. In fact, our data for the first time demonstrates that inhibiting InAT axis attenuates colorectal CSCs growth in a manner specific to inhibition of the MIB pathway. These findings are of high translational relevance as InAT axis is one of the most commonly deregulated pathways in colorectal cancer [32]. We have unequivocally demonstrated the importance of inhibition of the MIB pathway in IGF-1R attenuation mediated anti-CSCs effects at both phenotypic (spheroid formation) and molecular (CSC markers expression) levels. Moreover, two parallel approaches to inhibit IGF-1R, pharmacologic inhibition with a tyrosine kinase inhibitor as well as genetic knockdown, confirmed the significance of MIB downregulation as a specific and central mechanism of its anti-CSC effect. More importantly, our data point to a specific inhibition of the MIB pathway distal to IPP synthesis at nodal point(s) (e.g. IDI-1) distinct from that targeted by statins, a specific inhibitor of HMGCR, and bisphosphonates that target FDPS.

While the precise mechanism of specific downregulation of the MIB pathway by IGF-1R inhibition remains to be elucidated, our data points to regulation of the MIB pathway gene transcription as a potential mechanism since changes in mRNA expression of the MIB pathway genes proportionately coincided with changes in their protein levels. In this regard, our findings point to a potential role of AKT-mTOR signaling pathway downstream of IGF-1R in regulating the MIB pathway. In fact, we found that the active form of SREBPs (1&2) were significantly upregulated following IGF-1 treatment and downregulated by OSI-906. Previously, regulation of SREBP2 cleavage in AKT specific manner by IGF1/IGF-1R signaling was demonstrated in non-neoplastic cells [28, 29]. Hence, downregulation of IGF-1R-AKT-mTOR-SREBP2 axis might explain broader inhibition of the MIB pathway genes following IGF-1R inhibition. In fact, inhibition of mTOR with rapamycin inhibited colonosphere formation in a manner dependent on suppression of the MIB pathway and also prevented IGF-1 induced activation of the MIB pathway. Thus mTOR is a critical regulator of the MIB pathway downstream of IGF-1R. A recent report suggests that MIB and the cholesterol biosynthesis pathway was most enriched in raptor knockdown Treg cells, further supporting a specific regulation of the MIB pathway by the mTORC1 complex [33]. Moreover, mTOR can directly regulate transcription by associating with transcriptional machinery that regulates metabolic genes [34]. Hence, it is conceivable that the InAT axis might also regulate the MIB pathway in a specific manner. However, these postulates remain to be proven in carefully designed experiments and will be examined at length in the future studies.

Although, specific inhibitors of protein prenylation have resulted in some clinical benefits [35, 36], their development has been staggered due to concerns with side effects [37]. On the other hand, a few classes of FDA-approved drugs such as statins and nitrosylated bisphosphonates, which are widely used clinically have shown encouraging anticancer effects in pre-clinical and early phase clinical studies [38-42]. Plethora of evidence suggests that statins as well as oral bisphosphonate use is associated with reduced risk of several cancers including colorectal cancer [38, 40]. Moreover, statins and bisphosphonate treatment results in sensitization to chemotherapy in breast, colorectal cancer and/or leukemia [40, 42, 43]. One of the limitations of the statin therapy is feedback upregulation of SREBPs which mediate increased expression of the MIB pathway genes, resulting in restricting the MIB pathway inhibition by statins [22, 44]. This limitation can potentially be overcome by combining it with agents that inhibit feedback cleavage and activation of SREBPs, such as dipyridamole [22]. As a proof of principle, a combination of atorvastatin and dipyridamole synergistically induced apoptosis in multiple myeloma and acute myelogenous leukemia cells by dampening sterol-feedback loop [22]. Our own data clearly suggest that IGF-1R inhibition significantly downregulates mature form of (cleaved and transcriptionally active) SREBPs, raising a possibility that it could also dampen sterol-feedback loop induced by statin therapy and thus synergize with statins in inhibiting colon CSCs. More importantly, IGF-1R inhibitors (and/or mTOR inhibitors) might also synergize with statins or bisphosphonates to attenuate colon CSCs by targeting the MIB pathway at different nodes resulting in maximum inhibition of the MIB pathway. In fact, combination of statins and bisphosphonates has shown to be effective in pre-clinical models of multiple myeloma [45]. Additionally, a recent study suggested that growth of PC-3 prostate cancer cells in co-culture with mice calvariae was inhibited to a greater extent by a combination of IGF-1R inhibitor and simvastatin compared to either agent alone [46]. However, no mechanism was provided in the latter study to support the observed effects [46]. Carefully designed studies should be carried out in colonic CSCs in earnest to examine therapeutic synergism between inhibitors of IGF-1R pathway and the MIB pathway. Overall, we believe that our novel findings on regulation of the MIB pathway by IGF-1R provide a platform for immediately testable hypotheses of high translational relevance for the treatment of colorectal cancer in particular and cancer in general.

## MATERIALS AND METHODS

### Ethics statement

Investigation has been conducted in accordance with the ethical standards and according to the Declaration of Helsinki and according to national and international guidelines and has been approved by the authors' institutional review board.

### Cell culture & transfection

HCT-116 human colon cancer cells were kindly gifted by Dr. Majumdar (Wayne State University). HT-29, WiDR and COLO-320DM human colon cancer cells were obtained within 6 months directly from ATCC (Manassas, VA, USA). These cells were maintained in 10cm tissue cultured treated plates (USA Scientific, Ocala, FL) as monolayer in Dulbecco's Modified Eagle Medium: Nutrient Mixture F-12 (DMEM / F-12) (Gibco, Cat #11320-033) supplemented with 10% fetal bovine serum (FBS) (Gibco, Cat # 10438-026), and 1X Antibiotic-Antimycotic liquid (AA) (Cat # 15240, Gibco). The cells were passaged using trypsin containing ethylenediaminetraacetic acid (EDTA) (Gibco, Cat # 25300-054) before they reached 70% confluence. Stable cell lines of IGF-1R knockdown cells were generated by transfecting scrambled or IGF1-R shRNAs (Cat # TG320384, Origene) in HCT 116 with Lipofectamine 2000 (Cat #11668027, Invitrogen) using manufacturer's protocol, followed by puromycin selection.

### Colonosphere formation assay

For primary sphere formation, 100 cells per 100μl stem cell media (SCM) [DMEM:F12, 1X AA, 1X B27 supplement (Cat # 17504, Gibco), 20 ng/ml epidermal growth factors (Cat # E9644, Sigma-Aldrich), and 10 ng/ml fibroblast growth factor (Cat # 354060, BD Bioscience)] were plated in non-treated, low adhesion, 96 wells plate. Four hours after incubation, vehicle or drug/metabolite at desired concentrations (as described in the figure legend) were added to each well (at least in triplicates for each sample). On day 5, numbers of spheres ranging from 50-150 micrometer in diameter were counted using a phase contrast microscope and percent inhibition was calculated compared to control.

### Secondary and tertiary colonosphere assay

For secondary colonospheres, the 96-well plate of primary spheres was centrifuged at 1000 rpm for 1 min and the supernatant was removed. Spheres that settle at the base of the plate were trypsinized with 20 micro liter/well/and single cell suspension was prepared using vigorous mechanical dissociation. The numbers of viable cells were counted with 1:5 ratio of cell: trypan blue and then re-plated at 100cells/100 microliter/well in SCM media in a low adhesion plate. No further treatment with vehicle or drug was performed. Numbers of spheres were counted as above on Day 5. The same method was repeated for tertiary spheres.

### Extreme limiting dilution analysis

Extreme limiting dilution analysis (ELDA) was performed as described by Hu and Smyth. We have used this method in a previous publication [25]. Briefly, single cell suspension obtained from adherent cells were plated at a concentration of 128, 64, 32, 16, 8, 4, 2 and 1 cell per 100 μl SCM in 96-well ultra-low attachment plates and incubated for five days. After five days, the numbers of wells showing colonospheres were counted. The frequency of spheroid forming cells in a particular cell type was determined using ELDA web tool at http://bioinf.wehi.edu.au/software/elda.

### Western blotting analysis

Western blot analysis was performed according to the standard protocol described previously [27]. Briefly, HCT 116 and HT-29 cells were plated in SCM in a low adhesion 6-well plate. Treatment with vehicle or drug (as described in the figure legend) was added to the appropriate time frame on day 4. Cells were harvested (as described in the figure legend) and lysed in a lysis buffer containing 20 mM Sodium phosphate buffer pH7.4, 100 mM NaCl, 2 mM EDTA pH 8, 1% IGEPAL CA-630, 2.5 mM Sodium orthovanadate, 1mM PMSF, protease inhibitor cocktail (Roche, NJ, USA) and phosphatase inhibitor cocktail 2 and 3 (Sigma-Aldrich, USA) at a final pH of 8. Following centrifugation at 10,000 g for 15 min at 4°C, protein concentration of the supernatant was determined using the Pierce BCA protein assay kit (Thermo Scientific). 50μg of protein was separated by SDS-PAGE and transferred to PVDF membranes (Bio-Rad, Hercules, CA). The membranes were blocked with Tris buffered saline containing 0.1% Tween-20 (TBST) and 5% skim milk powder for 1h followed by overnight incubation with primary antibody (dilution 1:1000) at 4°C. Protein expression of phospho-IGF-I Receptor β (Tyr1135) (DA7A8) (3918, Cell Signaling), IGF-I Receptor β (111A9) (3018, Cell Signaling), phospho-Akt (Thr308) (C31E5E) (2965, Cell Signaling), Akt (pan) (C67E7) (4691, Cell Signaling), phospho-mTOR (Ser2448) (D9C2) XP (5536, Cell Signaling), mTOR (7C10) (2983, Cell Signaling), phospho-p70 S6 Kinase (Thr389) (108D2) (9234, Cell Signaling), p70 S6 Kinase (49D7) (2708, Cell Signaling), phospho-4E-BP1 (Thr70) (9455, Cell Signaling), 4E-BP1(53H11) (9644, Cell Signaling), CD44 (156-3C11) (3570, Cell Signaling), GAPDH (D16H11) XP (5174, Cell Signaling); HMGCR Antibody (C-1) (sc-271595, Santa Cruz Biotech), SREBP-1 Antibody (K-10) (sc-367, Santa Cruz Biotech), SREBP-2 Antibody (H-164) (sc-5603, Santa Cruz Biotech), MVK Antibody (H-300) (sc-366285, Santa Cruz Biotech); monoclonal mouse anti-IDI1 antibody (ab55317, Abcam), polyclonal rabbit anti-FDPS antibody (ab38854, Abcam); CD133/2 (293C3) antibodies, human (clone: 293C3) (130-090-854, Miltenyi Biotec), anti-LGR5 TRUEMAB antibody clone 2A2 (TA503316, Origene) and anti-MYC (06-340, Millipore) following incubation with appropriate secondary antibodies, protein bands were visualized using Supersignal west pico chemiluminescent substrate (Cat # 34080, Thermo Scientific) and imaged with LAS-3000 imaging system (FUJIFILM). Densitometry was analyzed by AIDA image analyzer software (Raytest, Germany) and the results were calculated as relative intensity compared to control. All experiments were performed at least three times.

### Immunoblotting analysis of tumor tissue

Snap frozen tumor tissue were homogenized in a lysis buffer (20 mM Tris-HCl,150mM NaCl,1 mM EDTA,1% Triton X-100 pH7.5), containing a protease inhibitor cocktail and phosphatase inhibitor cocktails 1 and 2. Lysates were cleared by centrifugation (10 minutes at 14,000 × g), diluted with 8% β-ME containing 5X SDS sample buffer and boiled. SDS-polyacrylamide gel electrophoresis and western blotting were performed by standard protocols as described above with equal amount of total protein (50 μg) per lane.

### Real-time PCR analysis

Total RNA was isolated using the mirVana™ miRNA Isolation Kit (Cat # AM1560, Life Technologies, Grand Island, NY) according to the manufacturer's protocol. 1 μg total RNA was reverse transcribed using First-Strand cDNA synthesis kit for Real-Time PCR using hexamer reverse primer (Cat # 75780, Affymetrix, Santa Clara, CA). Q-PCR was performed using RT² SYBR® Green qPCR mastermix (Cat # 330520, QIAGEN, Valencia, CA) in a 7500 Fast real time machine (Applied Biosystem, Grand Island, NY). Relative expressions of mRNA were calculated using ΔΔCT methods using GAPDH as control.

List of primers used in the study –

Forward primer CD133- 5′ GGA CCC ATT GGC ATT CTC 3′
Reverse primer CD133- 5′CAG GAC ACA GCA TAG AAT AAT C 3′
Forward Primer CXCR4 - 5 ‘ ACT ACA CCG AGG AAA TGG GCT 3′
Reverse Primer CXCR4 - 5 ‘ CCC ACA ATG CCA GTT A AG A AGA 3′
Forward primer LGR5 – 5′ CTC CCA GGT CTG GTG TGT TG 3′
Reverse primer LGR5 – 5′ GAG GTC TAG GTA GGA GGT GAA G 3′
Forward primer C-MYC – 5′ GGC TCC TGG CAA AAG GTC 3′
Reverse primer C-MYC – 5′ AGT TGT GCT GAT GTG TGG AGA 3′
Forward primer NANOG – 5′ CTC GTA TTT GCT GCA TCG TAA TG 3′
Reverse primer NANOG – 5′ CAC TCG GTG AAA TCA GGG TAA A 3′
Forward primer OCT-4 – 5′ GGG AGA TTG ATA ACT GGT GTG TT 3′
Reverse primer OCT-4 – 5′ GTG TAT ATC CCA GGG TGA TCC TC 3′
Forward Primer IGF1R - 5 ‘TCG ACA TCC GCA ACG ACT ATC 3′
Reverse Primer IGF1R - 5 ‘CCA GGG CGT AGT TGT AGA AGA G 3′
Forward Primer IGF1 - 5 ‘GGA GCT GTG ATC TAA GGA GGC 3′
Reverse Primer IGF1 - 5 ‘GGA CAG AGC GAG CTG ACT T 3′
Forward Primer IGF2 - 5 ‘GGA GAC GTA CTG TGC TAC CC 3′
Reverse Primer IGF2 - 5 ‘CTG CTT CCA GGT GTC ATA TTG G 3′
Forward Primer ACAT1 - 5 ‘ATG CCA GTA CAC TGA ATG ATG G 3′
Reverse Primer ACAT1 - 5 ‘GAT GCA GCA TAT ACA GGA GCA A 3′
Forward Primer HMGCS1 - 5 ‘CAT TAG ACC GCT GCT ATT CTG TC 3′
Reverse Primer HMGCS1 - 5 ‘TTC AGC AAC ATC CGA GCT AGA 3′
Forward Primer HMGCR - 5 ‘CTC CAG TAC CTA CCT TAC 3′
Reverse Primer HMGCR - 5 ‘GCT GCT GGC ACC TCC A 3′
Forward Primer MVD - 5 ‘GGA CCG GAT TTG GCT GAA TG 3′
Reverse Primer MVD - 5 ‘CCC ATC CCG TGA GTT CCT C 3′
Forward Primer MVK - 5 ‘CCT TTC GGA AGG ACA TGA TCC 3′
Reverse Primer MVK - 5 ‘TCT CCG TGT GTC ACT CAC CA 3′
Forward Primer IDI-1 - 5 ‘AAC ACT AAC CAC CTC GAC AAG C 3′
Reverse Primer IDI-1 - 5 ‘AGA CAC TAA AAG CTC GAT GCA A 3′
Forward Primer IDI-2 - 5 ‘GAC TGG GTT GAC AGG CGT C 3′
Reverse Primer IDI-2 - 5 ‘GTC GGC ACC AAT AAC CTT ATC AT 3′
Forward Primer FDPS - 5 ‘TGT GAC CGG CAA AAT TGG C 3′
Reverse Primer FDPS - 5 ‘GCC CGT TGC AGA CAC TGA A 3′

### RNA isolation from xenograft samples

Total RNA was isolated from the snap frozen tumor tissue using mirVana™ miRNA Isolation Kit. Briefly, xenograft tissue was re-suspended with 700 μl lysis/binding buffer and homogenized with QIAshredder column (Cat # 79654, QIAGEN, Valencia, CA). Total RNA was extracted with phenol/chloroform and purified according to manufacturer's protocol and integrity of purified RNA was confirmed with the spectrophotometer. Q-PCR was performed as described above.

### Gene microarray and analysis

Gene microarray was performed in duplicates and initial analysis was performed by Affymetrix GeneChip Service, LS Sciences, LLC. Full analysis was performed for two samples: HT-29 monolayer cell and HT-29 spheroids. Raw data, metadata, .CEL and .CHP were deposited in the NCBI GEO database, Accession number: GSE65433. Total RNA was isolated using the mirVana™ miRNA Isolation Kit according to the manufacturer's protocol and RNA intergrity was confirmed on the Nanodrop ND-1000 and analyzed with Agilent 2100 bioanalyzer. The samples were amplified and labeled using the Affymetrix 3′IVT labeling kit (Lot NO. 1010021) and hybridized with the GeneChip® Human Genome U133 Plus 2.0 Array for 16 hr. After hybridization, samples were washed and stained with the Affymetrix fluidics station 450. The arrays were scanned with the Affymetrix 3000 7G plus scanner. Dat and Cel files were obtained by the AGCC software, and the CHP files were generated with mas5 method by Affymetrix Expression Console. The data was imported into the Agilent GeneSpring GX software for further analysis. The data obtained through GeneChip® scanning was analyzed using Affymetrix® Microarray Suit Software 5.0. The genes whose expression was significantly altered by 2-fold in either direction were subjected to Ingenuity Pathways Analysis (Ingenuity® Systems, www.ingenuity.com).

### Flow cytometry analysis

Human colon cancer HCT 116 cells grown in adherent condition, serum starved overnight followed by treatment with vehicle or IGF1 (100 ng/ml) for 48 hours, were trypsinized and single cells were re-suspended at one million/ml in PBS buffer. Cells were incubated with conjugated antibody for 30 minutes at 4°C and washed once with PBS buffer prior to analysis. Following antibody and dilution were used: CD133/1 (AC133)-APC human (1:33 dilution) (Cat # 130-090-826, Miltenyi Biotec, Auburn, CA), CXCR4 (anti-mouse CD184)-PE conjugated clone 2B11 (Dilution 1:50) (Cat # 12-9991-82, eBioscience, San Diego, CA), and Anti-LGR5 mouse mAb, clone 2A2, PE conjugated (Dilution 1:50) (Cat # TA400001, Origene, Rockville, MA). Cell sorting was performed using FACS Aria™ II High-Speed Cell Sorter (BD Biosciences, San Jose, CA) and data were analyzed using FCS express 4 flow research edition (De-Novo Software Los Angeles, CA).

### *In vivo* xenograft assay

All experiments involving animals were approved by animal component of the research protocol (ACORP) according to VA McGuire Medical Center (Richmond, VA) guideline. One million HCT116 suspended in 50% BD Matrigel Matrix Growth Factor Reduced, Phenol Red-Free (356231, BD Bioscince) (in 50 μL sterile PBS) were injected into two sites on both flanks of 6-week-old NOD-SCID mice (Taconic Farms, Germantown, NY). Once tumors reached an average volume of 200 mm^3^ (Day 9) animals were randomly assigned to two groups that were gavaged with 200 μL saline or OSI-906 (25 mg/kg), as shown in the Fig. 6A for 21-days. Tumor measurements were made three time a week with Vernier calipers, and tumor volume was calculated using the formula: V = (W(2) × L)/2, where V=volume in mm^3^, W and L= width and length in mm. At the end of the 21-day post treatment (Day 30), the animals were sacrificed and the tumor tissue were chopped and digested with 400 μg/ml Collagenase Type IV (Cat # 07909, STEMCELL Technologies, Vancouver, BC). Single cell suspension was filtered with 70 μm cell strainer (BD Biosciences, San Jose, CA) and stained with PE conjugated LGR5 and PE conjugated CXCR4 antibody (1:50 dilution) and surface expression was analyzed with Aria-BD FACSAria™ II High-Speed Cell Sorter (BD Biosciences, San Jose, CA).

### Apoptosis assay in the xenograft tissue

Mice were sacrificed and tissue was snap frozen in liquid nitrogen using O.C.T. Compound (Sakura Finetek USA Inc.) and stored at −80C till further use. 5 micron sections of xenograft tissues were stained using ApopTag Peroxidase *In Situ* Apoptosis detection kit (Millipore, USA), as per the manufacturers protocol. Images were acquired on a Nikon TI-U fluorescent phase contract microscope. The number of TUNEL-positive cells was counted in ten random high-power fields (80x), and the average number of TUNEL-positive cells was determined for each condition tested.

### Statistical analysis

All data are expressed as means ± SEM unless otherwise indicated. The results were analyzed using the unpaired, two-tailed Student's t-test. *p < 0.05; **p < 0.005 was designated as the level of significance unless specified otherwise.

## SUPPLEMENTARY MATERIAL FIGURES



## Abbreviations

4EBP1: eIF-4E-binding protein 1
AA: Antibiotic-Antimycotic liquid
acetyl-CoA: Acetyl Coenzyme A
AKT: Protein kinase B
CD133: Prominin-1/Cluster of differentiation133
CD44: Cluster of differentiation 44
C-MYC: Myc proto-oncogene protein
CSC: Colorectal cancer stem cells
CXCR4: C-X-C chemokine receptor type 4
DCAMKL1: doublecortin and CaM kinase-like 1
DMAPP: Dimethylallyl pyrophosphate
DMEM/F12: Dulbecco's Modified Eagle Medium: Nutrient Mixture F-12
Dual (hi): CD133(hi)/CXCR(hi)
Dual (low): CD133(low)/CXCR4(low)
EDTA: ethylenediaminetraacetic acid
ELDA: Extreme limiting dilution analysis
FDPS: Farnesyl diphosphate synthase
FPP: Farnesyl pyrophosphate
GAPDH: Glyceraldehyde 3-phosphate dehydrogenase
HMGCR: 3-hydroxy-3-methyl-glutaryl-CoA reductase
HMGCS1: 3-hydroxy-3-methyl-glutaryl-CoA synthase
HPF: High power field
IC50: half maximal inhibitory concentration
IDI: Isopentenyl-diphosphate delta isomerase
IGF1: Insulin-like growth factor-1
IGF2: Insulin-like growth factor-2
IGF-1R: Insulin-like growth factor-1 receptor
IGF-1R KD: IGF-1R Knock down
InAT: Insulin-like growth factor-1 receptor / Protein kinase B / mammalian target of rapamycin
IPP: Isopentenyl pyrophosphate
LGR5: Leucine-rich repeat-containing G-protein coupled receptor 5
MAPK: Mitogen-activated protein kinases
MCF: Michigan Cancer Foundation
MIB: Mevalonate-isoprenoid biosynthesis
mTOR: mammalian target of rapamycin
mTORC1: mammalian target of rapamycin complex 1
MVA: Mevalonic acid
MVK: Mevalonate kinase
NANOG: Nanog homeobox
N-BP: nitrosylated-bisphophonates
OCT-4: octamer-binding transcription factor 4
p70S6K: p70S6 kinase
PI3K: Phosphatidylinositol-4,5-bisphosphate 3-kinase
PTEN: phosphatidylinositol-3,4,5-trisphosphate 3-phosphatase
RAF: proto-oncogene c-RAF;
SCM: Stem cell media
SP: Side population
SREBP: Sterol regulatory element binding proteins
TKI: Tyrosine kinase inhibitor
TUNEL: Terminal deoxynucleotidyl transferase dUTP nick end labeling
