# Supplementary material for: Inhibition of insulin-like growth factor receptor/AKT/mammalian target of rapamycin axis targets colorectal cancer stem cells by attenuating mevalonate-isoprenoid pathway in vitro and in vivo
Source: Oncotarget. 2015 Mar 29;6(17):15332–47. doi: 10.18632/oncotarget.3684 (PMC4558155; doi:10.18632/oncotarget.3684)
Supplement: Supplementary file 1 [file oncotarget-06-15332-s001.pdf]

Inhibition of insulin-like growth factor receptor/AKT/mammalian target of rapamycin axis targets colorectal cancer stem cells by attenuating mevalonate-isoprenoid pathway *in vitro* and *in vivo*

Supplementary Material

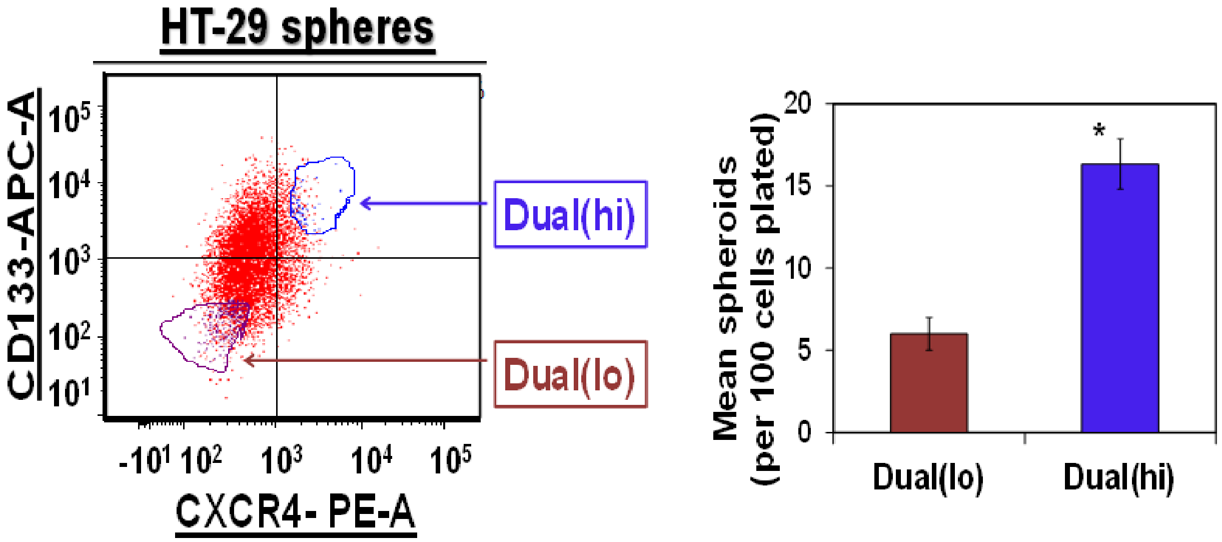

Supplementary Figure 1: Increased sphere formation in HT-29 sorted Dual(hi) cells. Florescence assisted sorting analysis in HT-29 spheroids demonstrates increase colonosphere forming ability in dual(hi) compared to dual(lo) cells. Data are presented as mean ± SD (n = 3). *p < 0.05; **p < 0.005

A. Top canonical pathways enriched in spheroids

Pathway	Enrichment ratio (number of genes significantly altered/total number of genes in the pathway)	P-value
Superpathway of Cholesterol Biosynthesis	0.400	<0.001
Cholesterol Biosynthesis I	0.462	<0.001
Cholesterol Biosynthesis II (via 24,25-dihydrolanosterol)	0.462	<0.001
Cholesterol Biosynthesis III (via Desmosterol)	0.462	<0.001
Mevalonate Pathway	0.417	<0.001
IGF-1 Signaling	0.2	<0.01
Her-2 Signaling	0.2	<0.05

B. Expression of Superpathway of Cholesterol Biosynthesis genes in spheroids

Symbol	Entrez Gene Name	Fold Change
ACAT2	acetyl-CoA acetyltransferase 2	2.839
CYP51A1	cytochrome P450, family 51, subfamily A, polypeptide 1	2.491
FDFT1	farnesyl-diphosphate farnesyltransferase 1	5.140
HMGCR	3-hydroxy-3-methylglutaryl-CoA reductase	2.374
HMGCS1	3-hydroxy-3-methylglutaryl-CoA synthase 1 (soluble)	7.489
IDI1	isopentenyl-diphosphate delta isomerase 1	4.514
MSMO1	methylsterol monooxygenase 1	2.939
MVD	mevalonate (diphospho) decarboxylase	3.973
NSDHL	NAD(P) dependent steroid dehydrogenase-like	2.112
SC5D	sterol-C5-desaturase	3.131
SQLE	squalene epoxidase	2.508

Supplementary Figure 2: Gene microarray analysis in HT-29 cells. A Gene microarray analysis was performed in HT-29 cells grown as monolayer and spheroid (enriched in CSCs) using Affymetrix® platform and data were analyzed through the use of Ingenuity Pathways Analysis (Ingenuity® Systems, www.ingenuity.com). (A) List of top canonical pathways showing highly significant enrichment ($p<0.05$) in spheroids compared to monolayer controls are represented in a tabular form. The enrichment ratio

indicates proportions of genes in a given pathway that are significantly altered in the spheroids compared to monolayer controls. Two related pathways: superpathways of cholesterol biosynthesis, and mevalonate pathway (highlighted in blue) as well as IGF-1 signaling pathway (highlighted in red) were amongst the top canonical pathway enriched in spheroids. **(B)** Table lists relative expression of genes in the superpathway of cholesterol biosynthesis, top canonical pathway, in HT-29 spheroids compared to monolayer controls. The genes that are common amongst the two related pathways of superpathway of cholesterol biosynthesis and mevalonate pathway (panel A, highlighted in blue) are highlighted in red font.

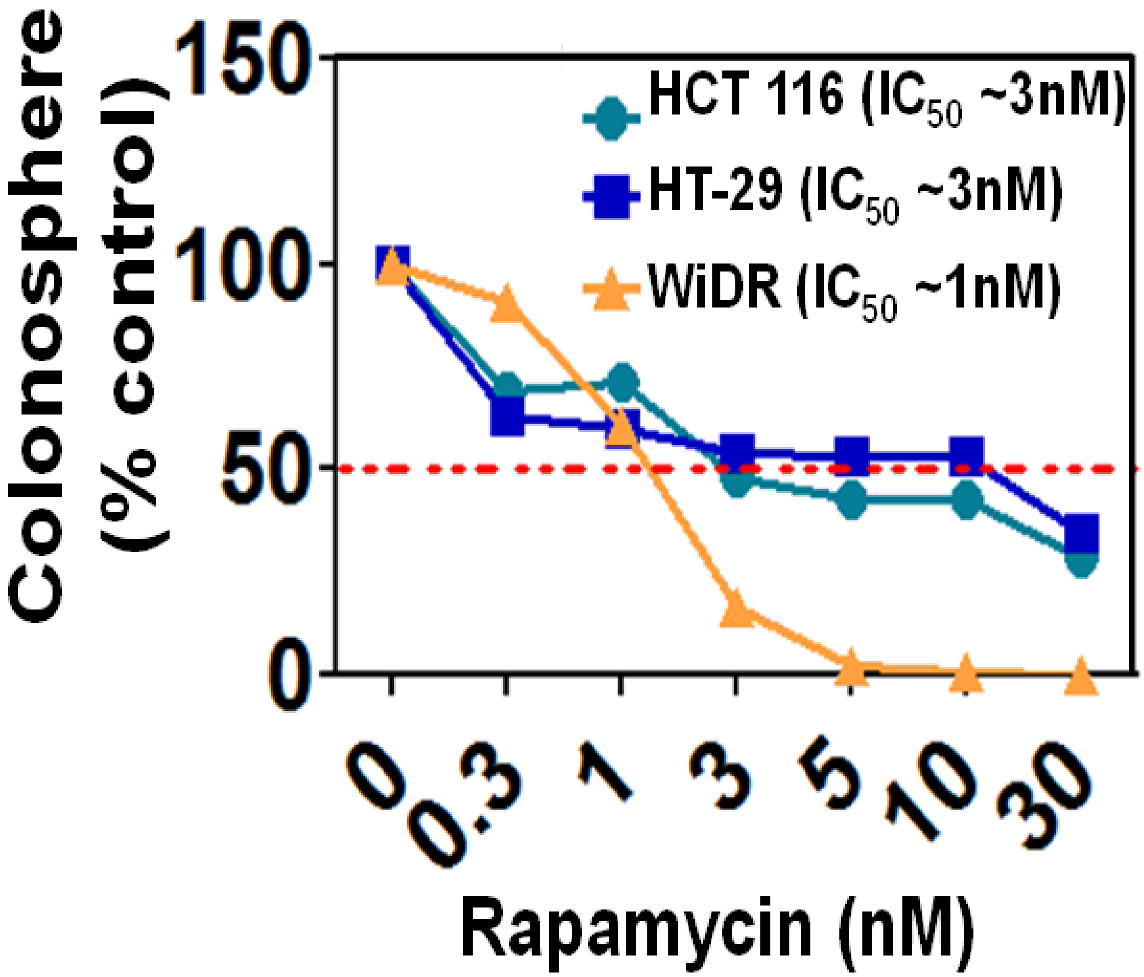

Supplementary Figure 3: Rapamycin inhibits spheroid growth in a dose dependent fashion. Dose response curve shows inhibition of primary colonosphere formation in three colon cancer cell lines (HCT-116, HT-29 and WiDR) treated with Rapamycin, an mTOR inhibitor, with an IC_{50} ranging from 1 nM -3 nM compared to vehicle treated controls. Data are presented as mean ± SD (n = 3).

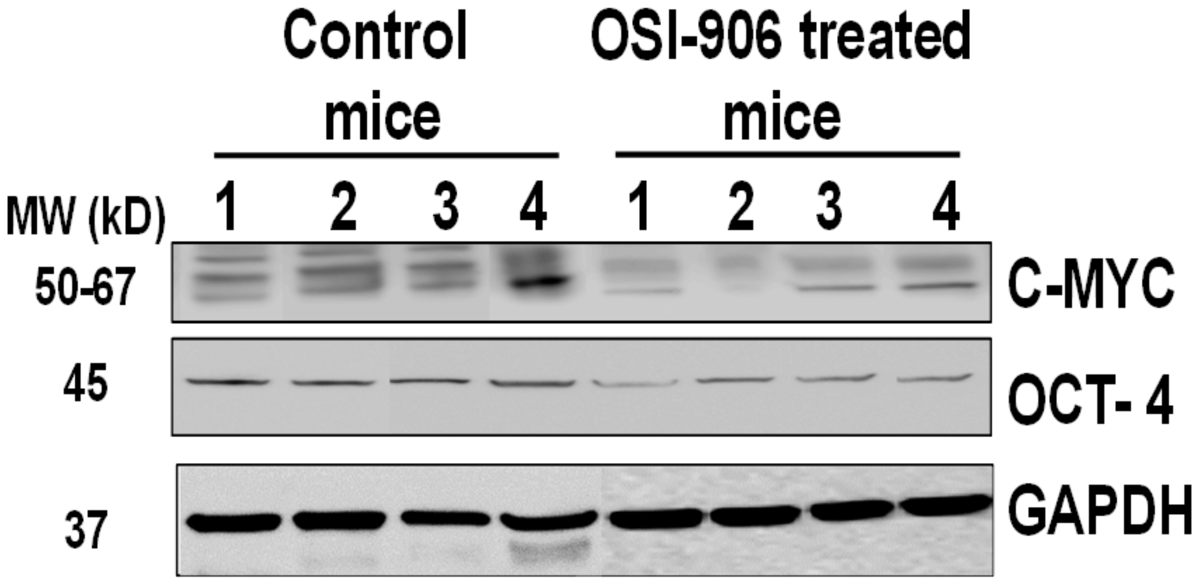

Supplementary Figure 4: Self-renewal marker expression in OSI-906 xenografts. Immunoblots show decreased expression of self-renewal factor (C-MYC and OCT-4) in four OSI-906 treated xenografts compared to vehicle controls. GAPDH is used as a loading control. Numbers under the blot represent relative densitometry values. Data are presented as mean ± SD (n = 3).
